# State of Medicine in Turkey

**Published:** 1843-01

**Authors:** 


### State of Medicine in Turkey.

An hospital for instruction has been recently opened in Constantinople in the medical school of Galataserai. It consists of a medical, surgical, and ophthalmic clinic, each of which contains fifty beds. It is distinguished by its cleanliness, comfort, elegance, abundant funds, and good management, in which it may be fairly ranked with any similar European establishment. Its chief officer is Dr. Bernard, who contributed greatly to its establishment; and the second officer is Dr. Hermann, inspector of the military hospitals. The medical school possesses everything which such an institution requires; an anatomical, mineralogical, zoological, and physical museum, a clinical laboratory, botanic garden, library, hospital, and a well-arranged dissecting-room in which the bodies, even of Mussulmen, are dissected. All that is wanted is a midwifery institution. The great jealousy of the Turks is an important obstacle to its establishment, but it is hoped that this may in time be overcome.

*Allgemeine Medic. Central Zeitung. Juli* 20, 1842.

